# Emerging and Emerged Pathogenic *Candida* Species: Beyond the *Candida albicans* Paradigm

**DOI:** 10.1371/journal.ppat.1003550

**Published:** 2013-09-26

**Authors:** Nicolas Papon, Vincent Courdavault, Marc Clastre, Richard J. Bennett

**Affiliations:** 1 Université François-Rabelais de Tours, EA2106, Biomolécules et Biotechnologies Végétales, Tours, France; 2 Department of Microbiology and Immunology, Brown University, Providence, Rhode Island, United States of America; Duke University Medical Center, United States of America

## *Candida albicans* and Non-*albicans Candida* (NAC) Species Infections: General Information in Predisposing Conditions and Clinical Incidence

Many ascomycete yeast species from the *Candida* genus are widely distributed in nature and act as common saprophytic constituents of the normal human microflora. However, some of these fungal species can also become opportunistic pathogens following a transition from a commensal to a pathogenic phase, induced by alterations in the host environment. *Candida* species thereby rarely trigger infection in healthy people, but take advantage of a locally or systematically impaired immune system to proliferate in the host and cause diseases termed “candidiasis.” Such fungal infections can be subdivided into three major groups: cutaneous (skin and its appendages), mucosal (oropharyngeal, esophageal, and vulvovaginal) and systemic (bloodstream infections, i.e., candidemia and other forms of invasive candidiasis [IC]). While superficial candidiasis (cutaneous and mucosal) is often observed in AIDS patients, oropharyngeal thrush and vaginitis are more frequently seen in immunocompetent infants and adult women, respectively. Candidemia and IC are common in cancer patients or in transplant individuals following immunosuppression. Candidiasis currently represents the fourth leading cause of nosocomial infections, at 8% to 10%, and mortality due to systemic candidiasis remains high, ranging from 15% to 35% depending on the infecting *Candida* species [1].

Although *Candida albicans* remains the most frequently isolated agent of candidiasis, non-*albicans Candida* (NAC) species now account for a substantial part of clinical isolates collected worldwide in hospitals. NAC species of particular clinical importance include *Candida glabrata*, *Candida tropicalis*, *Candida parapsilosis*, and *Candida krusei* (synonym: *Issatchenkia orientalis*), as well as the less-prominent species *Candida guilliermondii*, *Candida lusitaniae*, *Candida kefyr*, *Candida famata* (synonym: *Debaryomyces hansenii*), *Candida inconspicua*, *Candida rugosa*, *Candida dubliniensis*, and *Candida norvegensis* (Table 1). A complementary set of about 20 opportunistic NAC species is also known, but exhibits lower isolation rates [2].

**Table 1:** Introducing characteristics of Candida species.

Species	Freq.^a^	Resistance^b^	Morphology^c^	Sex.^d^	Ploidy^e^	Genome sequence^f^	Molecular tools available^g^
*C. albicans*	63.8% (49–68)		Yeast, Pseudohyphae, Hyphae	+	Diploid	Available	Selectable markers, Reporter genes, Regulatable systems
*C. glabrata*	11.3% (7–21)	Polyenes (+), Azoles (+)	Yeast, Pseudohyphae	−	Haploid	Available	Selectable markers, Reporter genes, Regulatable systems
*C. tropicalis*	7.2% (5–13)		Yeast, Pseudohyphae, Hyphae	+	Diploid	Available	Selectable markers, Reporter genes
*C. parapsilosis*	6.0% (4–14)	Echinocandins (+)	Yeast, Pseudohyphae	−	Diploid	Available	Selectable markers, Reporter genes, Regulatable systems
*C. krusei (I. orientalis)*	2.4% (1–4)	Polyenes (+), Azoles (+++)	Yeast, Pseudohyphae	+	Diploid	Available	
*C. guilliermondii*	0.7% (0.1–2)	Echinocandins (+), Azoles (+)	Yeast, Pseudohyphae	+	Haploid	Available	Selectable markers, Reporter genes
*C. lusitaniae*	0.6% (0.5–0.6)	Polyenes (→ +++)	Yeast, Pseudohyphae	+	Haploid	Available	Selectable markers
*C. kefyr* (*K. marxianus*)	0.5% (0.3–0.6)		Yeast, Pseudohyphae	+	ND	Available	Selectable markers
*C. famata (D. hansenii)*	0.3% (0.1–0.5)	Azoles (+)	Yeast, Pseudohyphae	+	Haploid	Available	Selectable markers, Reporter genes
*C. inconspicua*	0.2% (0.1–0.5)	Azoles (+++)	Yeast, Pseudohyphae	+	ND		
*C. rugosa*	0.2% (0.1–1)	Polyenes (+++), Azoles (+++)	Yeast, Pseudohyphae	−	Haploid		Selectable markers
*C. dubliniensis*	0.1% (0.1–0.2)		Yeast, Pseudohyphae, Hyphae	+	Diploid	Available	Selectable markers, Reporter genes, Regulatable systems
*C. norvegensis*	0.1% (0.02–0.1)	Azoles (+++)	Yeast, Pseudohyphae	+	ND		

a from reference [2], Freq.: frequency of isolation (range).

b from reference [1], (+++): strong primary resistance; (+): moderate primary resistance; (→ +++): strong secondary resistance (acquired).

c from reference [14].

d from reference [6], Sex.: sexual or parasexual reproduction; ND: unknown.

e from reference [21].

f from references [19], [21]–[24].

g from references [5], [10].

## Trends in Species Distribution and Antifungal Susceptibility of NAC Species

Global surveillance programs (e.g. SENTRY and ARTEMIS) provide a tremendous amount of data regarding global trends in various aspects of NAC candidiasis including geographical variation in the frequency of species, distribution by specimen type and patient age, as well as changes in the antifungal susceptibility of collected NAC isolates [2].

An overview of the literature from the last four decades highlights an important fact: Due to the growing size of the population at special risk (due to neutropenia, immunosuppression, metabolic dysfunction, and anticancer chemotherapy), candidiasis remains a persistent public health problem, and the proportion of NAC species among *Candida* isolates recovered from patients is increasing. Whereas NAC species accounted for 10%–40% of all systemic candidiasis from 1970 to 1990, this proportion reached 35%–65% in the last two decades [3]. A recent ten-year analysis of the worldwide distribution of NAC species indicated that *C. glabrata* remains the most common NAC species and that *C. parapsilosis*, *C. tropicalis*, and *C. krusei* are also frequently isolated (Table 1). *C. guilliermondii* and *C. lusitaniae* have shown gradual emergence as a cause of invasive candidiasis, while *C. kefyr*, *C. famata*, *C. inconspicua*, *C. rugosa*, *C. dubliniensis*, and *C. norvegensis*, although rarely isolated, are now considered emerging NAC species, as their isolation rate has increased between 2- and 10-fold over the last 15 years [2].

Interestingly, significant geographic variation in the frequency of NAC species occurs. Among marked trends, *C. glabrata* is more prominent in North America than in Latin America. In addition, *C. tropicalis* is frequently isolated in Asia-Pacific and less often encountered in the rest of the world, whilst *C. parapsilosis* remains 3-fold more commonly recovered in North America than in Europe. Finally, *C. guilliermondii* and *C. rugosa* are more prominent in Latin America, and *C. inconspicua* and *C. norvegensis* in Europe [2] than in the rest of the world.

Antifungal compounds currently used to treat systemic candidiasis belong to three families: polyenes, azoles, and echinocandins. Most of the NAC species exhibit particular patterns of primary resistance or reduced susceptibility toward these antifungals (Table 1). For example, a high level of resistance toward azoles is well known for *C. krusei*, *C. inconspicua*, *C. rugosa*, and *C. norvegensis*, whereas *C. parapsilosis* and *C. guilliermondii* stand out due to their decreased susceptibility to echinocandins [4].

## A Particular Codon Usage in Most NAC Species Delays Development of Genetic Tools

Since the end of the last century, the clinical importance of NAC species has promoted research aimed at identifying molecular events underlying pathogenicity and antifungal resistance in these emerging yeasts. However, the development of genetic approaches in NAC species has been hindered by three main factors: (i) most pioneering studies during the early stages of the “pathogenic yeast genetics” field were carried out in *C. albicans*; (ii) the particular codon usage of most of *Candida* species has precluded the direct use of *S. cerevisiae* or bacterial molecular tools in these NAC species [5]; (iii) most pathogenic *Candida* species have limited modes of sexual reproduction unlike *S. cerevisiae* [6].

**Figure 1:**
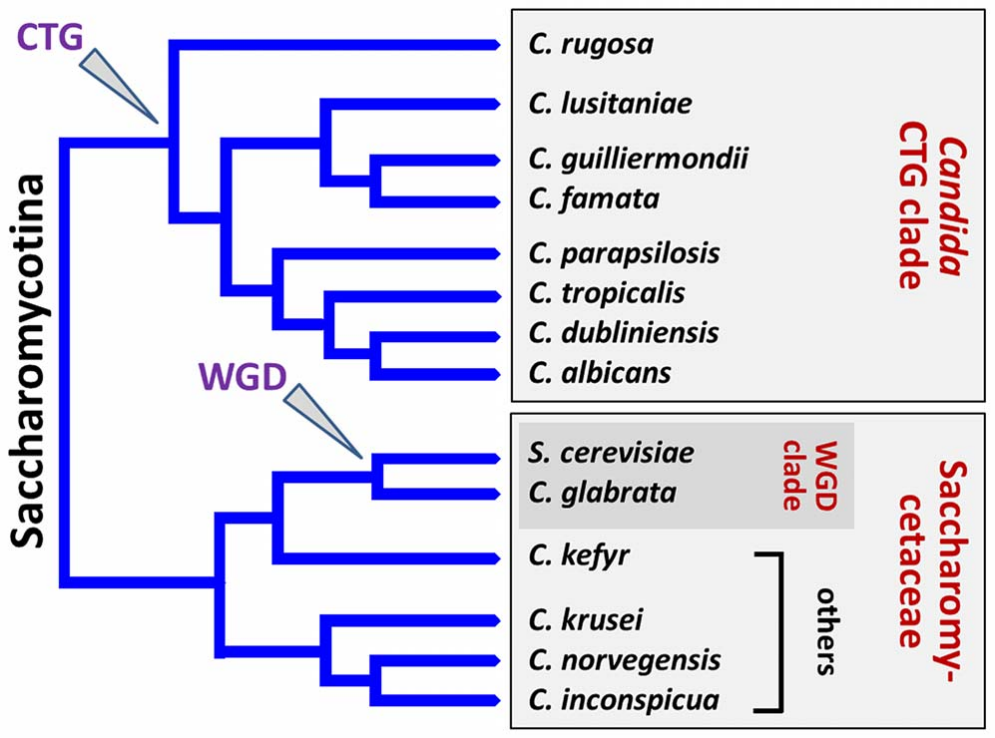
Schematic representation illustrating the phylogeny of NAC species. *C. tropicalis*, *C. parapsilosis*, *C. guilliermondii*, *C. lusitaniae*, *C. famata (D. hansenii)*, *C. rugosa*, and *C. dubliniensis* form part of the *Candida* CTG clade and translate CTG codons as serine instead of leucine. In contrast, *C. glabrata* and *C. kefyr* (*K. marxianus*) belong to the Saccharomycetaceae, with *C. glabrata* and *S. cerevisiae* falling within the “whole genome duplication” (WGD) clade. The remaining species *C. krusei* (*I. orientalis*), *C. inconspicua*, and *C. norvegensis* are probably closely related in the Saccharomycetaceae clade. The branch lengths are arbitrary.

Originally, the genus name *Candida* was attributed to yeast species able to form hyphae or pseudohyphae (Table 1) and for which no sexual spores were observed. Nevertheless, recent phylogenetic analysis has clarified that *Candida* species actually represent a polyphyletic group within the Saccharomycotina [7] (Figure 1). More precisely, *C. tropicalis*, *C. parapsilosis*, *C. guilliermondii*, *C. lusitaniae*, *C. famata*, *C. rugosa*, and *C. dubliniensis* form part of the *Candida* CTG clade and translate CTG codons as serine instead of leucine. In contrast, *C. glabrata* and *C. kefyr* belong to the Saccharomycetaceae, with *C. glabrata* and *S. cerevisiae* falling within the whole genome duplication (WGD) clade. The remaining species *C. krusei*, *C. inconspicua*, and *C. norvegensis* are probably closely related in the Saccharomycetaceae clade, which could give insights into their common resistance toward azole antifungals.

During the late 1990s, *C. glabrata* genetics was by far the most advanced of the NAC species due to its haploid status, its classical codon usage (allowing the direct use of *S. cerevisiae* tools), and its high frequency of isolation in hospitals [8]. Genetic studies of CTG clade species expanded in the 2000s and focused on the development of molecular tools, as well as transformation procedures, due to the biotechnological potential of several *Candida* yeasts (*C. guilliermondii*, *C. famata*, *C. tropicalis*, and *C. rugosa*) as well as clinical incidence (*C. dubliniensis* and *C. parapsilosis*) [5], [9]. Specifically, drug-resistant markers and reporter genes (encoding fluorescent protein variants, luciferase, or beta-galactosidase) were adapted by changing CTG codons to allow their functionality in this particular clade [5] (Table 1).

## Mechanisms Underlying Antifungal Resistance, Virulence, and Morphological Transitions in NAC Species: Is *Candida albicans* the Rule or the Exception?

*C. albicans* genetics, with the construction and phenotypical analysis of targeted mutant strains since 1994, has provided a foundation for understanding fundamental processes in pathogenic yeasts [10]. Intense research in *C. albicans* from the end of the 20^th^ century shed light on the molecular mechanisms involved in drug resistance [11], biofilm formation [12], adherence [13], yeast-hyphal switching and its role in virulence [14], and sexual mating [15], [16]. *C. albicans* has therefore become the model yeast for investigating the multiple factors controlling the host–pathogen interaction. As a result, *C. albicans* biology is now the paradigm for *Candida* research in the medical mycology community.

In response to the clinical emergence of NAC species, research programs were initiated to further understand these opportunistic yeasts. The first studies highlighted marked differences in behavior between different *Candida* species. This included stress adaptation [17], which may come from the fact that each species has independently evolved to promote survival in their respective natural niches and their specific host. It must also be kept in mind that each *Candida* species displays specific traits such as ploidy, sexual behavior (if any) [6], and morphology [14] (Table 1). These could directly impact their ability to adapt to the host's response, to disseminate in the organism, and to develop resistance mechanisms to antifungals during treatments.

Due to the lack of genetic and molecular resources, researchers have often assumed that if a yeast species is related to another yeast species, the underlying molecular and cellular mechanisms must also be closely related. However, even within a clade, the genetic distance between any two NAC species is often larger than the genetic distance between humans and some fishes [18]. Therefore, in no way should it be argued that *C. albicans* makes the rules for all NAC species. As a corollary, in future investigations, the biology of each *Candida* species should continue to be addressed on a case-by-case basis.

## Perspectives: Genome Resources and Postgenomic Technologies Dedicated to NAC

A large range of rapidly evolving genomic and postgenomic approaches, including genome sequences and gene expression data, have recently enhanced the understanding of *Candida* yeasts pathogenicity.

The first published genomes of *Candida* species were *C. glabrata* in 2003 (alongside the *C. famata* genome sequence) [19], followed by *C. albicans* [20] in 2004, which has further strengthened the prominent role of *C. albicans* and *C. glabrata* in the field. In January 2005, the Broad Institute Fungal Genome Initiative, in collaboration with the Wellcome Trust Sanger Institute, made available the sequences of five CTG clade genomes, including *C. tropicalis*, *C. parapsilosis*, *C. dubliniensis*, *C. guilliermondii*, and *C. lusitaniae* [21], [22]. Finally, genome sequences of *C. kefyr* (teleomorph *Kluyveromyces marxianus*) [23] and *C. krusei* [24] were recently published. These genome resources have provided new insights into gene family evolution within *Candida* species and identified gene families enriched in the most common pathogenic NAC species [21]. This area of research is further supported by the creation of databases dedicated to genome annotation, including gene ontology browsers specializing in metabolic pathways, virulence, and morphogenesis [25]. These bioinformatics tools provide an accurate annotation of NAC genome sequences and give precious help to future *Candida* gene evolutionary analyses.

Postgenomic technologies have also emerged to support the *Candida* research field. Quantitative transcriptional profiling strategies (e.g. RNA-Seq, microarray) currently allow the active screening of genes commonly or specifically required for pathogenicity, morphogenesis, and antifungal resistance in multiple *Candida* species [26]–[28].

Thanks to the growing number of yeast genome sequences available, as well as the utilization of postgenomic approaches, the palette of newly identified pathogenicity-related genes in NAC species is now predicted to increase rapidly. However, efforts need to continue toward the development of classical molecular tools dedicated to each pathogenic NAC species to further analyze the function of large numbers of uncharacterized genes. This is an essential prerequisite for the identification of new fungal targets and the subsequent development of novel antifungal drugs.
